# Comparison of Time Series Methods and Machine Learning Algorithms for Forecasting Taiwan Blood Services Foundation's Blood Supply

**DOI:** 10.1155/2019/6123745

**Published:** 2019-09-17

**Authors:** Han Shih, Suchithra Rajendran

**Affiliations:** ^1^Department of Industrial and Manufacturing Systems Engineering, University of Missouri, Columbia, MO 65211, USA; ^2^Department of Marketing, University of Missouri, Columbia, MO 65211, USA

## Abstract

**Purpose:**

The uncertainty in supply and the short shelf life of blood products have led to a substantial outdating of the collected donor blood. On the other hand, hospitals and blood centers experience severe blood shortage due to the very limited donor population. Therefore, the necessity to forecast the blood supply to minimize outdating as well as shortage is obvious. This study aims to efficiently forecast the supply of blood components at blood centers.

**Methods:**

Two different types of forecasting techniques, time series and machine learning algorithms, are developed and the best performing method for the given case study is determined. Under the time series, we consider the Autoregressive (AUTOREG), Autoregressive Moving Average (ARMA), Autoregressive Integrated Moving Average (ARIMA), Seasonal ARIMA, Seasonal Exponential Smoothing Method (ESM), and Holt-Winters models. Artificial neural network (ANN) and multiple regression are considered under the machine learning algorithms.

**Results:**

We leverage five years worth of historical blood supply data from the Taiwan Blood Services Foundation (TBSF) to conduct our study. On comparing the different techniques, we found that time series forecasting methods yield better results than machine learning algorithms. More specifically, the least value of the error measures is observed in seasonal ESM and ARIMA models.

**Conclusions:**

The models developed can act as a decision support system to administrators and pathologists at blood banks, blood donation centers, and hospitals to determine their inventory policy based on the estimated future blood supply. The forecasting models developed in this study can help healthcare managers to manage blood inventory control more efficiently, thus reducing blood shortage and blood wastage.

## 1. Introduction

Blood performs several important functions in the human body such as transporting oxygen, carrying supplements to our cells, disposing ammonia, carbon dioxide, and other waste items. Four of the most critical elements are the red blood cells (RBC), white blood cells (WBC), plasma, and platelets [1]. The American Red Cross reported that over 35,000 RBC units, 10,000 plasma units, and 7,000 platelet units are required day-to-day within the US [2]. Due to the short shelf life of blood components, hospitals and blood centers are faced with the challenge of maintaining appropriate inventory levels to avoid outdating and shortage.

Managing blood supply and demand is the core part of the healthcare supply chain system as blood plays a very crucial role in saving human lives. Blood supply forecasting is essential for making supply chain decisions, such as donor drive scheduling, vehicle routing policies, and inventory management, at blood centers and hospitals. Accurate forecasts of the timing and amount of future blood requests have been considered as the key inputs to donor recruitment decision making and inventory control. It is important to gather data for several years to forecast monthly demand and to recognize seasonality in demand [3–6]. Lestari et al. [7] indicated that the forecasting can predict the data trend observed and future demand for blood components.

## 2. Literature Review

Several studies have leveraged time series forecasting techniques for predicting the blood demand at hospitals and blood centers. For instance, Pereira [8] investigated and evaluated the autoregressive integrated moving average (ARIMA) model and Holt-Winters exponential smoothing model to predict monthly demand for red blood cell transfusions at a tertiary care. While these methods focused on using time series forecast, Bosnes et al. [9] used the statistical regression technique for the forecast of blood donor arrivals at the blood bank of Oslo and found that the most important factors among 18 explanatory variables were: donor age, time from making an appointment to arriving at the drive, contact methods used, number of prior donations, and donor no-show rate. Fortsch and Khapalova [10] introduced numerous practical methods to predict future demand of blood. Several forecasting models, including the naïve, exponential smoothing, moving average, and time series decomposition, were tested using the daily demand data from a blood center that were obtained for January 2006 to December 2012. They also compared the performance of these methods with an autoregressive moving average (ARMA) model. The results revealed that the ARMA forecasting model performed better for eight out of nine time series model settings. Similarly, Khaldi et al. [11] explored the capabilities of employing machine learning algorithms such as the artificial neural network (ANN) model to predict future demand for blood.

## 3. Materials and Methods

As discussed earlier, the study aims to develop effective forecasting methods to predict the supply of RBCs using two different techniques: time series forecasting methods and machine learning algorithms.

### 3.1. Time Series Forecasting

This section discusses the seven time series forecasting methods used in this study.

#### 3.1.1. Autoregressive (AUTOREG) Model [12, 13]

The AUTOREG procedure estimates and forecasts linear regression models for time series data when the errors are autocorrelated. The autoregressive model regresses the value of the series at time *t* (*Y*_*t*_) on the values during the time periods *t* − 1, *t* − 2,…, *t* − *p*.  The mathematical formula is expressed as follows:

(1) $$\begin{array}{c} \;Y_{t}=\alpha_{0}+\alpha_{1}Y_{t-1}+\alpha_{2}Y_{t-2}+\cdots +\alpha_{p}Y_{t-p}+\varepsilon_{t}, \end{array}$$

where *α*_0_, *α*_1_, *α*_2_,…, *α*_*p*_ are the linear regression coefficients, *Y*_*t*_ is the forecasted value at time *t*, and *ε*_*t*_ is the random error variable and is generally assumed to have a normal distribution with mean 0 and variance *σ*^2^ (i.e., normal (0, *σ*^2^)).

#### 3.1.2. Autoregressive Moving Average (ARMA) Models [12–14]

ARMA model is one of the basic tools in time series modeling. Suppose the time series *Y*_1_, *Y*_2_,…, *Y*_*t*_ is a stationary stochastic process time series, the expression ARMA (*p*, *q*) represents the model with autoregressive order of *p* and moving-average order of *q*. This model is a combination of the AR (*p*) and MA (*q*) models, where AR (*p*) is written as *Y*_*t*_=*a*+∅_1_*Y*_*t*−1_+∅_2_*Y*_*t*−2_+⋯+∅_*p*_*Y*_*t*−*p*_+*ε*_*t*_ and MA (*q*) is written as *Y*_*t*_=*b* − *θ*_1_*ε*_*t*−1_ − *θ*_2_*ε*_*t*−2_ − ⋯−*θ*_*q*_*ε*_*t*−*q*_+*ε*_*t*_.

As in the AUTOREG model, *Y*_*t*_ is the observation value at time *t*. The ARMA (*p*, *q*) process is generally written as follows:

(2) $$\begin{array}{c} Y_{t}=c+\overset{p}{\underset{i=1}{\sum }}\emptyset_{i}Y_{t-i}-\overset{q}{\underset{i=1}{\sum }}\theta_{i}\varepsilon_{t-i}+\varepsilon_{t}, \end{array}$$

where *a*, *b*, and *c* are constants, *ε*_*t*_ is the random error variable and is generally assumed to have a normal distribution with mean 0 and variance *σ*^2^; ∅_1_, ∅_2_,…, ∅_*p*_ are the autoregressive coefficients to be estimated, and *θ*_1_, *θ*_2_,…, *θ*_*q*_ are the moving average coefficients to be estimated.

#### 3.1.3. Autoregressive Integrated Moving Average (ARIMA) Model [12–14]

The ARIMA (autoregressive integrated moving average) approach was made popular by Box–Jenkins models [11]. The ARIMA procedure is functioning as a linear combination of its current values, past values, past errors, and past values of other time series (predictor time series) to predict a future response value in a time series.

With time series nonstationary behavior, the above ARMA (*p*, *q*) model can be extended and written using difference which is defined as: *Y*_*t*_ − *Y*_*t*−1_=(1 − *B*)*Y*_*t*_= ∇*Y*_*t*_, where *t* is the index of time, *Y*_*t*_ is time series {*Y*_*t*_ :  1 ≤ *t* ≤ *n*}  at time *t*, and *B* is the backward shift operator, which means that *B* has the effect of shifting the data back one period (i.e., *BY*_*t*_=*Y*_*t*−1_).

#### 3.1.4. Seasonal ARIMA Model [12, 13, 15, 16]

Seasonal ARIMA model is written with the general expression ARIMA (*p*, *d*, *q*)(*P*, *D*, *Q*)_*s*_. The symbol *p* is the order of the nonseasonal autoregressive component, *d*  is the order of the differencing, *q* is the order of the nonseasonal moving-average process, *P* is the order of the seasonal autoregressive part, *D*  is the order of the seasonal differencing, *Q*  is the order of the seasonal moving-average process, and *s* is the duration of the seasonal cycle.

Let *Y*_*t*_ be a dependent time series {*Y*_*t*_ :  1 ≤ *t* ≤ *n*} at time *t*, then the mathematical formula for the seasonal ARIMA model is expressed as follows:

(3) $$\begin{array}{c} {\left(1-B\right)}^{d}{\left(1-B^{s}\right)}^{D}Y_{t}=µ+\frac{\theta \left(B\right)\theta_{s}\left(B^{s}\right)}{\varphi \left(B\right)\emptyset_{s}\left(B^{s}\right)}\varepsilon_{t}, \end{array}$$

where *μ* is the constant mean, *B*^*s*^ is the seasonal backward shift operator, ∅_*s*_(*B*^*s*^)=1 − ∅_*s*,1_(*B*^*s*^) − ⋯− ∅_*s*,*P*_(*B*^*sP*^) is the seasonal autoregressive component, and *θ*_*s*_(*B*^*s*^)=1 − *θ*_*s*,1_(*B*^*s*^) − ⋯−*θ*_*s*,*Q*_(*B*^*sQ*^) is the seasonal moving-average component.

#### 3.1.5. Seasonal Exponential Smoothing Model [12, 13, 15, 16]

In the seasonal exponential smoothing method (ESM), the equation of forecast value at time *t*+*k* (*Y*_*t*+*k*_) is given by

(4) $$\begin{array}{c} Y_{t+k}=L_{t}+S_{t-p+k}. \end{array}$$

The smoothing equations are as follows:

(5) $$\begin{array}{c} L_{t}=\alpha \left(X_{t}-S_{t-p}\right)+\left(1-\alpha \right)L_{t-1}, \end{array}$$

(6) $$\begin{array}{c} S_{t}=\gamma \left(X_{t}-L_{t}\right)+\left(1-\gamma \right)S_{t-p}, \end{array}$$

where *X*_*t*_ is given observation at time *t*, and *α* and *γ* are the level and seasonal smoothing parameters, respectively, *L*_*t*_ is the estimated level component at time *t*, *S*_*t*_ is the estimated seasonal component at time *t*, and *p* is the periods after which the seasonal cycle repeats itself.

#### 3.1.6. Multiplicative Holt-Winters Model [12, 13, 15, 16]

The Holt-Winters model, also known as the triple exponential smoothing, applies three types of exponential smoothing to the time series—value, trend, and seasonality. The model equation for the Holt-Winters method can be either additive or multiplicative model. In this section, we present the multiplicative Holt-Winters model, whereas Section 3.1.7 presents the additive model.

The mathematical formula relevant to a time series with a trend and constant seasonal component using the Holt-Winters additive technique has the forecast at time *t*+*k* (*Y*_*t*+*k*_) given by following equation:

(7) $$\begin{array}{c} Y_{t+k}=\left(L_{t}+kT_{t}\right){\text{SI}}_{t+k-p}. \end{array}$$

The smoothing equations are given using the following equations:

(8) $$\begin{array}{c} L_{t}=\alpha \left(\frac{X_{t}}{SI_{t-p}}\right)+\left(1-\alpha \right)\left(L_{t-1}+T_{t-1}\right), \end{array}$$

(9) $$\begin{array}{c} T_{t}=\beta \left(L_{t}-L_{t-1}\right)+\left(1-\beta \right)T_{t-1}, \end{array}$$

(10) $$\begin{array}{c} {\text{SI}}_{t}=\gamma \left(\frac{X_{t}}{L_{t}}\right)+\left(1-\gamma \right){\text{SI}}_{t-p}, \end{array}$$

where *X*_*t*_ is given observation at time *t*,  *α*,  *β*,   and *γ* are the level, trend, and seasonal corresponding constants, respectively, *L*_*t*_ is the estimated level at time *t*, *T*_*t*_ is the estimated trend at time *t*, SI_*t*_ is the seasonality index at time *t*, and *p* is the periods after which the seasonal cycle repeats itself.

#### 3.1.7. Additive Holt-Winters Model [12, 13, 15, 16]

In this section, we present the additive Holt-Winters Model.

For the additive model, the forecasted supply estimate for time *t*+*k* is given by the following equation:

(11) $$\begin{array}{c} Y_{t+k}=L_{t}+kT_{t}+S_{t-p+k}. \end{array}$$

The estimates of level, trend, and seasonal factors for additive model equations are given using the following equations:

(12) $$\begin{array}{c} L_{t}=\alpha \left(Y_{t}-S_{t-p}\right)+\left(1-\alpha \right)\left(L_{t-1}+T_{t-1}\right), \end{array}$$

(13) $$\begin{array}{c} T_{t}=\beta \left(L_{t}-L_{t-1}\right)+\left(1-\beta \right)T_{t-1}, \end{array}$$

(14) $$\begin{array}{c} S_{t}=\gamma \left(Y_{t}-L_{t}\right)+\left(1-\gamma \right)S_{t-p}. \end{array}$$

### 3.2. Machine Learning Algorithms

Machine learning is a technology exploring the algorithms to analyze a set of data, learn from the insights gathered, and make predictions on data [17]. For the blood supply forecasting, we leverage the two most widely used machine-learning techniques, artificial neural network and regression.

#### 3.2.1. Artificial Neural Networks (ANN)

ANN is a reinforcement learning method that is an adaptation of biological neural network. The network consists of several nodes that are distributed across numerous layers, and each layer is connected to its previous and subsequent layers within the network [17]. These interconnected elements work closely to process information that they receive from the nodes of the previous layers and transfer them to the next layer based on the sigmoid function. They are particularly useful for modeling complex relationships in high-dimensional data or where the relationship between the input and output variables is not easy to understand [17].

#### 3.2.2. Multiple Regression

Multiple regression is another class of problem in machine learning that is trying to predict a continuous value of a variable instead of a class unlike in classification problem [17]. Linear regression with ordinary least square is one of the classic machine learning algorithms in this domain. The mathematical formula for the regression model is represented as follows:

(15) $$\begin{array}{c} Y=\beta_{0}+\beta_{1}X_{1}+\cdots +\beta_{n}X_{n}+\varepsilon , \end{array}$$

where *Y* is the response variable, *X*_*n*_ is an independent variable, *β*_0_ is the intercept, *β*_*i*_ is the slope of the coefficient *X*_*i*_ (both *β*_0_ and *β*_*i*_ are unknown coefficients to be estimated by the model), and *ε* is the error variable.

### 3.3. Evaluation of the Different Methods

We use four different measures of forecast errors for evaluating the model performance and the accuracy of the methods; they are MAE, MSE, BIAS, and MAPE [12, 15, 18].

Assume *X*_1_, *X*_2_,…, *X*_*n*_ are actual data and *F*_1_, *F*_2_,…, *F*_*n*_ are forecasted data, and then the *n* values of forecast errors, *e*_1_, *e*_2_,…, *e*_*n*_, are given by *e*_1_=*F*_1_ − *X*_1_,  *e*_2_=*F*_2_ − *X*_2_,…, *e*_*n*_=*F*_*n*_ − *X*_*n*_.

- Mean absolute error (MAE): it measures the average significance of the forecast errors, where all individual errors have equal weights:

(16) $$\begin{array}{c} \text{MAE}=\frac{1}{n}\overset{n}{\underset{i=1}{\sum }}\left|e_{i}\right|. \end{array}$$

(b) Mean squared error (MSE): it also measures the significance of the forecast errors, and larger errors get penalized more due to squaring:

(17) $$\begin{array}{c} \text{MSE}=\frac{1}{n}\overset{n}{\underset{i=1}{\sum }}e_{i}^{2}. \end{array}$$

(c) BIAS: this is an indication of whether the forecast is overestimating or underestimating the actual supply over the forecast horizon:

(18) $$\begin{array}{c} \text{BIAS}=\overset{n}{\underset{i=1}{\sum }}e_{i}. \end{array}$$

(d) Mean absolute percentage error (MAPE): it measures the relative significance of forecasting errors in percentage terms:

(19) $$\begin{array}{c} \text{MAPE}=\frac{1}{n}\overset{n}{\underset{i=1}{\sum }}\left|\frac{e_{i}}{X_{i}}\right|\times 100. \end{array}$$

## 4. Results

### 4.1. Data Collection

The historical supply data for five years from 2013 to 2017 are first gathered from the health records. The summary statistics are given in Table 1.

From Table 1, it is observed that the average blood supplies of the weekdays for each year are steady. Also, we can see that Monday supply is very high, Thursday and Friday supplies are quite high, Tuesday and Wednesday supplies are moderate, and Saturday and Sunday supplies are significantly lower.

### 4.2. Time Series Forecasting Results

After running the seven different time series models discussed in Section 3.1 and obtaining the forecasts, we evaluate them using the error measures given in Section 3.3, and the results are presented in Table 2. It is clear that Seasonal ARIMA Model, Seasonal Exponential Smoothing Method, and Multiplicative Holt-Winters Model yield minimal error measures. Hence, we conclude that, under the time series methods, these three models are best forecasting the blood supply for the case study data under consideration.

### 4.3. Machine Learning Algorithm Results

The performance of the machine learning algorithms is compared in Table 3. For this particular dataset, results show that regression is a better predictor of the blood supply, nevertheless, the power of the results using regression is quite low (*R*^2^ = 63.71%).

Therefore, regression is used to predict the supply for the first week of January 2018 as shown in Table 4. A summary of the results obtained under the time series method and regression is given in Table 4.

Clearly from the results, we can infer that there is not a single method that predicts the supply accurately, and hence we recommend using the average value of the forecasts obtained under these four methods for estimating the future supply [15, 19–21].

## 5. Discussion

This study focusses on predicting the supply of red blood cells for Taiwan Blood Services Foundation (TBSF) [22], a nongovernmental and nonprofit organization. So far, more than seven million citizens have donated blood in Taiwan through this foundation (which accounts for over 25% of the total population of Taiwan) [23]. Currently, blood centers at TBSF do not have a proper blood forecasting system, and some blood centers face blood shortage problems as a result to lack of accurate forecasting of blood supply. This paper focusses on developing a blood supply forecasting decision support tool for TBSF using time series and machine learning algorithms. The accurate forecasting models will enable TSBF to make good blood supply chain management planning decisions, such as when to collect blood from donors, how much units to collect, proper assignment of the workforce for collecting blood in donor drives, and blood component testing process. Upon accurately forecasting the future supply using the methods discussed in this study, inventory models can then be developed to make decisions on the number of units to order and time between orders.

There are some limitations on forecasting methods. Accuracy of forecasting could be affected by various factors. If there are some unknown variable(s) that could cause some of the fluctuations in the data, then it will be more difficult to forecast unless there are known explanatory variable(s) accounting for the variations. Blood supply forecasting is vital for blood supply chain decisions, and they have to be updated as more reliable information becomes available. Hence, after appropriate forecasting methods are selected, it is important to continuously monitor the forecast accuracy.

## Figures and Tables

**Table 1:** 2013–2017 TBSF weekly supply summary statistics.

Year	Day	Average	Min.	Max.	Standard deviation	Coefficient of supply variation (%)
2013	Sunday	188	32	461	84	44.68
	Monday	1,523	173	1,928	287	18.84
	Tuesday	820	154	1,558	200	24.39
	Wednesday	961	327	1,606	254	26.43
	Thursday	1,127	299	1,596	282	25.02
	Friday	1,039	458	1,956	263	25.31
	Saturday	135	43	462	68	50.37

2014	Sunday	174	31	456	82	47.13
	Monday	1,525	688	2,324	351	23.02
	Tuesday	858	327	1,935	253	29.49
	Wednesday	857	168	1,474	210	24.50
	Thursday	1,238	80	2,048	304	24.56
	Friday	1,013	84	2,027	314	31.00
	Saturday	138	31	587	103	74.64

2015	Sunday	200	39	531	126	63.00
	Monday	1,504	850	2,636	303	20.15
	Tuesday	850	495	1,421	200	23.53
	Wednesday	855	1	1,461	252	29.47
	Thursday	1,381	139	1,923	309	22.38
	Friday	1,025	197	1,450	253	24.68
	Saturday	164	31	660	122	74.39

2016	Sunday	204	31	542	99	48.53
	Monday	1,497	162	2,073	331	22.11
	Tuesday	855	372	1,572	239	27.95
	Wednesday	862	146	1,264	199	23.09
	Thursday	1,439	547	2,643	319	22.17
	Friday	1,060	81	2,058	301	28.40
	Saturday	146	55	490	69	47.26

2017	Sunday	201	50	522	116	57.71
	Monday	1,445	212	1,964	324	22.42
	Tuesday	888	355	1,508	238	26.80
	Wednesday	888	272	1,656	224	25.23
	Thursday	1,383	502	1,846	273	19.74
	Friday	1,159	57	2,061	312	26.92
	Saturday	192	41	679	100	52.08

**Table 2:** Error measures obtained under the seven time series models.

Error	Method						
	AUTOREG	ARMA	Basic ARIMA	Seasonalized ARIMA	Seasonalized ESM	Multiplicative Holt-Winters	Additive Holt-Winters
MAE	215	449	600	160	158	159	159
MSE	88,031	288,002	577,197	57,235	57,111	57,111	57,189
BIAS	−383	−20,578	754	−5,575	−7,338	−8,507	−15,056
MAPE	94.50	227	224	80	81	81	80

**Table 3:** Performance of machine learning algorithms.

Statistics of fit	Artificial neural network	Regression
R-square	58.59%	63.71%

**Table 4:** Blood supply predictions using the best performing time series and machine learning methods.

Methods	Prediction						
	1/1/2018	1/2/2018	1/3/2018	1/4/2018	1/5/2018	1/6/2018	1/7/2018
Seasonalized ARIMA	1491	899	882	1301	1242	200	208
Seasonalized ESM	1480	901	883	1314	1232	200	210
Multiplicative Holt-Winters	1490	906	887	1308	1251	202	210
Regression	1458	1269	1088	951	779	589	410
Actual supply	979	1223	972	1354	721	263	203

## Data Availability

The data used to support the findings of this study have not been made available because they are confidential to the case study blood center and hospitals.
